# Potential benefits and limitations of machine learning in the field of eating disorders: current research and future directions

**DOI:** 10.1186/s40337-022-00581-2

**Published:** 2022-05-08

**Authors:** Jasmine Fardouly, Ross D. Crosby, Suku Sukunesan

**Affiliations:** 1 School of Psychology, UNSW Sydney, Sydney, NSW 2052 Australia; 2 Sanford Center for Biobehavioral Research, Sanford Research, Fargo, ND USA; 3 Swinburne University of Technology, Melbourne, VIC Australia

**Keywords:** Machine learning, Artificial learning, Statistics, Eating disorder, Social media, Chatbot, Detection, Treatment, Ethical concerns

## Abstract

Advances in machine learning and digital data provide vast potential for mental health predictions. However, research using machine learning in the field of eating disorders is just beginning to emerge. This paper provides a narrative review of existing research and explores potential benefits, limitations, and ethical considerations of using machine learning to aid in the detection, prevention, and treatment of eating disorders. Current research primarily uses machine learning to predict eating disorder status from females’ responses to validated surveys, social media posts, or neuroimaging data often with relatively high levels of accuracy. This early work provides evidence for the potential of machine learning to improve current eating disorder screening methods. However, the ability of these algorithms to generalise to other samples or be used on a mass scale is only beginning to be explored. One key benefit of machine learning over traditional statistical methods is the ability of machine learning to simultaneously examine large numbers (100s to 1000s) of multimodal predictors and their complex non-linear interactions, but few studies have explored this potential in the field of eating disorders. Machine learning is also being used to develop chatbots to provide psychoeducation and coping skills training around body image and eating disorders, with implications for early intervention. The use of machine learning to personalise treatment options, provide ecological momentary interventions, and aid the work of clinicians is also discussed. Machine learning provides vast potential for the accurate, rapid, and cost-effective detection, prevention, and treatment of eating disorders. More research is needed with large samples of diverse participants to ensure that machine learning models are accurate, unbiased, and generalisable to all people with eating disorders. There are important limitations and ethical considerations with utilising machine learning methods in practice. Thus, rather than a magical solution, machine learning should be seen as an important tool to aid the work of researchers, and eventually clinicians, in the early identification, prevention, and treatment of eating disorders.

## Plain English Summary

Machine learning models are computer algorithms that learn from data to reach an optimal solution for a problem. These algorithms provide exciting potential for the accurate, accessible, and cost-effective early identification, prevention, and treatment of eating disorders, but this potential is just beginning to be explored. Research to date has mainly used machine learning to predict women’s eating disorder status with relatively high levels of accuracy from responses to validated surveys, social media posts, or neuroimaging data. These studies show potential for the use of machine learning in the field, but we are far from using these methods in practice. Useful avenues for future research include the use of machine learning to personalise prevention and treatment options, provide ecological momentary interventions via smartphones, and to aid clinicians with their treatment fidelity and effectiveness. More research is needed with large samples of diverse participants to ensure that machine learning models are accurate, unbiased, and generalisable to all people with eating disorders. There are limitations and ethical considerations with using these methods in practice. If accurate and generalisable machine learning models can be created in the field of eating disorders, it could improve the way we identify, prevent, and treat these debilitating disorders.

Eating disorders have severe and chronic impacts on the lives of sufferers and those who care for them [1]. These disorders typically begin in adolescence or early adulthood [2, 3], a time of upmost importance for the formation of long-term relationships and academic and career success. Thus, it is vital that we identify those at risk for, or suffering from, eating disorders early in life, before symptom severity changes the life course. Lifestyle modification and dissonance-based eating disorder prevention programs were recently found to be effective at reducing 54% to 77% of future eating disorder onset [4]. Although these results are encouraging, most prevention programs are in-person [4], making them difficult and costly to implement on a mass scale. Further, effective internet-based prevention programs have issues with adherence and dropout, and it is currently difficult to broadly disseminate the programs to those most at risk for eating disorders [4, 5]. Current approaches to identify eating disorder risk are primarily limited to validated questionnaires and clinical interviews [6]. Although these screening methods are effective, they are costly, time consuming, and burdensome to participants and/or the healthcare system. More importantly, they are typically administered on an individual basis to people who have already sought help from a health professional or those involved in eating disorder prevention programs, which has resulted in most people with an eating disorder not currently being screened [7, 8].

For those who do seek help for an eating disorder, waitlists are long and treatments are costly [1]. Response rates for even the best eating disorder treatments are modest [9–11], especially when considering long term outcomes [12]. Scholars have argued for the need for a personalised approach to treatment [11, 13], given research suggesting that treatment outcomes may vary based on individual characteristics [14, 15] and therapeutic alliance [16, 17]. However, further research is needed using large datasets to determine who will respond best to which treatments and we are far from implementing evidence-based personalised treatments for eating disorders on a mass scale. Thus, further innovations in the field of eating disorders are needed.

Machine learning (ML)—a form of artificial intelligence—are computer algorithms that learn from data to reach an optimal solution for a problem. Increases in computing power and digital storage capacity have given rise to the use of ML in research and healthcare settings. While ML is currently being utilised in physical healthcare to create automated approaches to diagnosis and treatment, in mental health, ML is primarily used in research rather than real-world settings [18]. Research on ML and eating disorders is just beginning to emerge [19]. This narrative review will explore current research and future potential benefits of ML to aid in the rapid and cost-effective detection, prevention, and treatment of eating disorders, and will highlight some limitations and ethical considerations with utilising these methods in practice.

## Machine learning

ML provides a wide suite of models for identifying patterns in data. The main aim of ML is to create a model that can make accurate predictions that are generalizable to different populations. Similar to traditional statistical models, ML models can be used to test hypotheses and make inferences about the data (i.e., inferential statistics), but they can also allow for flexible methods to maximise prediction and different methods of validation (i.e., predictive statistics) [20, 21]. Two common categories of ML models are supervised and unsupervised models [22]. Most models currently used in mental health research are supervised [23]. Supervised models are developed and tested on data that is labelled with the outcome. Supervised models can include pre-selected predictors based on existing literature and clinical experience and thus have some utility in hypothesis testing. Hypothesis-free approaches are also beneficial in identifying predictors that may be less intuitively known. Thus, supervised models with identifiable predictors can help generate future hypotheses and inform the development of theoretical models. Unsupervised models examine hidden and complex patterns in data that are unlabelled with the outcome and thus do not often facilitate human understanding of relevant predictors. Unsupervised learning is beneficial when human understanding is limited or biased within a domain. This approach leads to discovery of new patterns in data which are not possible to find using accepted methods.

When examining a small number of predictors, current research is mixed on whether ML outperforms traditional analytic approaches in predicting treatment outcomes in clinical settings [24], including those for eating disorders [25–27]. The main benefit of ML over traditional statistical models is the ability of ML to simultaneously examine large numbers (100s to 1000s) of multimodal predictors and their complex non-linear interactions [20]. Other benefits of ML methods include the emphasis on model parsimony, with techniques available to identify the simplest prediction models that don’t compromise on accuracy. ML methods also emphasise the importance of cross-validation [28], with data being separated into training and testing samples. Cross-validation is vital in psychology, especially when considering concerns around the replication of findings in the field, because it identifies whether a model has been overfit to the original (i.e., training) sample and whether it will generalise to new samples (i.e., testing samples) [21].

Although there are some ML techniques (e.g., regularisation, or testing the relative accuracy of models with and without specific predictors) that allow complex models to be more easily interpreted [21], highly complex and uninterpretable models are often found to make the most accurate predictions in many fields of psychology [29]. Thus, researchers will need to consider the extent to which they want to preference explanation or prediction within their data when deciding which ML or traditional statistical model best fits their research question. ML is not a replacement for statistical models but rather a technique to be used in conjunction with more traditional approaches to data analysis. There will always be the need to make interpretable and causal inferences within the field of eating disorders. However, there is also a need to make accurate and generalisable, yet perhaps uninterpretable, predictions. ML provides exciting potential to move the field forward and better predict eating disorder risk, prevention, and treatment outcomes.

## Implications for detection

In the field of eating disorders, supervised ML is primarily being used to predict people’s eating disorder status and to identify the most relevant predictors for eating disorders. Recent research has used ML to predict recurrent binge-eating behaviour [30] and eating disorder status [31–34] using cross-sectional surveys with validated measures of known eating disorder risk factors (see Table 1 for a summary of current research using survey predictors). Further, a longitudinal study used ML to predict future eating disorder outcomes among a sample of female patients over a two-year period from 33 self-report measures with 78% accuracy [26]. These studies provide initial evidence for the utility of ML to identify eating disorder status with relatively high accuracy (70–91%) using survey measures as predictors and help advance knowledge about the most relevant predictors of eating disorders, which have implications for prevention and treatment-seeking individuals. More longitudinal research is needed with larger samples of diverse participants to investigate the utility of different ML techniques to predict future eating disorder status using validated surveys. However, because these studies rely on self-report surveys as predictors, it is difficult and costly to implement these methods of screening on a mass scale.

**Table 1:** Research using ML to detect eating disorder risk via survey responses

Study	Sample	Predictors variables	Outcome variables	Best performing ML approach	Explanatory power of best performing model	Limitations
Buscema et al. [49]	172 females with a diagnosed ED	124 different variables: generic information, alimentary behaviour, eventual treatment and hospitalization, substance use, menstrual cycles, weight and height, hematochemical and instrumental examinations, psychodiagnostic tests	ED diagnosis	Feed forward neural networks	87%	Cross-sectional study Small sample
Forrest et al. [27]	191 adults with BED in a randomised controlled trial	Treatment condition, demographic information, baseline clinical characteristics	(1) Binge eating abstinence; or (2) reduction; (3) ED psychopathology; (4) perceived weight loss; and (5) actual weight loss	Elastic net	(1) 51%; (2) 4%; (3) 27%; (4) 12%; (5) 68%	Cross-sectional study Small sample
Haynos et al. [26]	415 female adults with a diagnosed ED, of which 320 completed measures at Year 1, and 277 completed measures at Year 2	Demographics, psychiatric treatment, ED symptoms, other psychiatric diagnoses and symptoms, self-esteem	(1) ED diagnosis; (2) objective binge eating; (3) compensatory behaviours; and (4) underweight BMI	Elastic net regularized logistic regressions	At year 1: (1) 62%; (2) 77%; (3) 88%; (4) 93% At year 2: (1) 61%; (2) 71%; (3) 85%; (4) 89%	Small sample
Krug et al. [33]	1402 adolescents and adults (92% female), with (n = 588) or without (n = 760) a diagnosed ED	Cross-cultural risk factors for EDs before the age of 12	(1) ED onset; (2) differential ED diagnoses	Penalised logistic regression (LASSO)	(1) 89%; (2) 70%	Cross-sectional study
Linardon et al. [30]	1341 adults (91% females), with (n = 512) and without (n = 829) recurrent binge eating	Intuitive eating behaviours, flexible restraint behaviours, rigid restraint behaviours, rigid restraint cognitions	Recurrent binge eating behaviour	Decision tree classification	70%	Cross-sectional study
Orru et al. [31]	107 females, with (n = 53) and without (n = 54) a diagnosed ED	Presence of manic/hypomanic and depressive symptoms, AN and BN symptoms	ED status	Naïve bayes	91%	Cross-sectional study Small sample
Ren et al. [34]	830 non-clinical young females	Psychological distress, eating inflexibility, body image inflexibility, body dissatisfaction, emotional overeating, loss of control overeating, body mass index	ED risk	Decision tree classification	85%	Cross-sectional study
Rosenfield and Linstead [32]	44 female young adults, with (n = 20) and without (n = 24) a previous diagnosis of AN	ED symptoms, psychosocial impairment, symptoms of autism spectrum disorder	ED status	K-means clustering	78%	Cross-sectional study Small sample

ED, eating disorder; AN, anorexia nervosa; BN, bulimia nervosa; BED, binge eating disorder; BMI, body mass index; ML, machine learning

Technology is an integral part of our lives and every swipe, click, like, post, purchase, and search can be stored as part of our ‘digital exhaust’. In 2016, more than 90% of the world’s data had been created in the past two years alone, and digital data is predicted to double in size every two years as internet use expands globally [35]. In a time of ‘big data’, there is vast potential to use ML to examine our digital data to make predictions about our current and future mental health. The advantages of using ML to analyse these data to detect risk are: (1) it does not require additional effort or burden to the individual; (2) screening can occur as soon as data is created; (3) it can be delivered across entire populations; and (4) it can identify those who are unaware of their risk and/or who may not seek help. Utilising ML on digital data could also help differentiate between specific eating disorders and other disorders with similar symptomologies and improve our understanding of the prevalence of these disorders within a population.

While there are many forms of digital data that can be examined (e.g., electronic health records, sensory data from smartphones/watches), most research in the field of eating disorders has focused on social media data. Social media is used from a young age [36] and posts provide access to naturalistic, first person accounts of users’ behaviour, thoughts, and feelings. Social media may be particularly useful for the identification of eating disorders because appearance-based images and comments are pervasive on social media and because eating disorder communities (whether pro-disorder or recovery) tend to gather on these platforms [37].

Pro-eating disorder content can be difficult to moderate on social media [38]. Two studies have used ML models to predict whether eating disorder content had been or would be removed on Instagram or Tumblr for violating community guidelines (see Table 2 for a summary of current research using ML to identify eating disorder content on social media). One study [39] using image and text content from Tumblr posts as predictors and using an unsupervised deep neural network ML model had a higher accuracy rate (89%) than the other study [40] using only text content from Instagram posts as predictors and a supervised model (69%). Further research is needed to determine whether the complexity of the predictors and/or the ML models improves their ability to identify potentially harmful eating disorder content on social media. However, current research suggests there may be utility in the use ML to rapidly remove potentially harmful or triggering eating disorder content on those platforms.

**Table 2:** Research using ML to detect eating disorder risk via social media and internet data

Study	Sample	Predictors variables	Outcome variables	Best performing ML approach	Explanatory power of best performing model	Limitations
Benítez-Andrades et al. [47]	494,025 posts containing ED-related words on Twitter	Posts with ED-related words	(1) Posts written by users who identify online as suffering from EDs; (2) posts that promoted having an ED; (3) informative posts; (4) scientific posts	Bidirectional encoder representations from transformer–based models (RoBERTa)	(1) 83%; (2) 89%; (3) 84%; (4) 94%	Outcome based on content of posts not validated measures of EDs Only identifies content of people who publicly acknowledge their ED on Twitter Specific predictors unknown
Chancellor et al. [40]	62,000 posts with removed pro-ED content or ED content remaining publicly available on Instagram	Combinations and frequencies of different ED hashtags and captions	Whether the post was removed or still publicly available	Logistic regression	69%	Only identifies content of people who publicly acknowledge their ED on Instagram
Chancellor et al. [41]	26 million posts from 100,000 users who post pro-ED content on Instagram	Mental illness severity (MIS; low, medium, high) in a user’s previous posts based on the content of hashtags	MIS (low, medium, high) based on the content of hashtags	Multinomial logistic regression	81%	MIS inferred from posts not validated measures
Chancellor et al. [39]	877,000 pro-ED photo posts shared on Tumblr, 569 of which were removed by Tumblr	Text, hashtag, and photo content from Tumblr posts	Whether the post would be/was removed by Tumblr for violating community guidelines	Deep neural network	89%	Specific predictors unknown
De Choudhury [37]	55,334 posts collected from 18,923 blogs on Tumblr who mentioned common ED and anorexia symptomatology tags	Social, affective, cognitive, and linguistic style expression in posts	(1) Whether a post shares any kind of anorexia related content; (2) Whether a post relates to the proana or the pro-recovery community	Support vector machine classifier	(1) 83%; (2) 81%	Outcome based on content of posts not validated measures of EDs Only identifies content of people who publicly acknowledge their ED on Tumblr
Hwang et al. [45]	185,950 posts and 3,528,107 comments from a weight management subcommunity on Reddit	4 types of emotional eating behaviours and 5 types of feedback based on Latent Dirichlet Allocation topic modelling method	Emotional eating diagnosis based on authors’ expertise	Stochastic gradient descent	91%	Outcome based on content of posts not validated measures of EDs
Sadeh-Sharvit et al. [48]	231 adult women on Prolific who contributed their internet browsing history over the past 6-months	Keywords related to EDs, daily visits to social media, fraction of searches on Google or Bing, activity rates, participant age	ED status (clinical/subclinical ED, high risk for an ED, or no ED) based on responses to validated surveys	GentleBoost	53%	Small sample size
Wang et al. [44]	119,825,361 posts on Twitter from 72,047 users, of which 1,797,239 posts were from 3380 users who self-identify with an ED on Twitter	User engagement and activity, posting preference, interaction diversity, psychometric properties of posts	ED status (ED or non-ED user)	Support vector machine	97%	ED status determined by self-identifying ED on Twitter Only identifies content of people who publicly acknowledge their ED on Twitter
Yan et al. [46]	4759 posts from 6 ED-related subcommunities on Reddit	Relationships between key words within the text of each post	Whether users need immediate mental health support for an ED based on expertise of two clinical psychologists	Logistic regression	96%	Required human coders with extensive expertise
Zhou et al. [43]	18,288 posts on Twitter with ED-related words	ED-related words in posts	ED-related topic clusters/themes	Correlation Explanation (CorEx) topic model	78%	Outcome based on content of posts not validated measures of EDs Only identifies content of people who publicly acknowledge their ED on Twitter
Zhou et al. [42]	123,977 posts on Twitter with ED-related words	Posts with ED-related words	(1) ED-relevant and ED-irrelevant posts; (2) ED-promotional and education and ED-laypeople posts	Convolutional neural network (CNN) and long short-term memory (LSTM)	(1) 89%; (2) 90%	Outcome based on content of posts not validated measures of EDs Only identifies content of people who publicly acknowledge their ED on Twitter

ED, eating disorder; ML, machine learning

ML has also been shown to be effective (78–97% accuracy) in identifying eating disorder content using certain hashtags and linguistic features relating to appearance, eating, and exercise from publicly available posts within eating disorder communities on different social media platforms [37, 41–47]. For example, one study examining pro-eating disorder content on Instagram was able to predict future mental illness severity with 81% accuracy over a seven month period [41], however it is important to note that severity was also predicted from content in the posts. This is a common limitation in the current literature, with most research inferring eating disorder status based on the social media posts themselves or based on human coding of posts for eating disorder risk, rather than participants’ responses to validated clinical surveys or interviews. The creation of accurate ML models that can predict eating disorder status based purely on social media posts may be an efficient and cost-effective method of screening in the future. However, those models first need to be created on large and diverse samples of people whose eating disorder status has been verified using validated clinical measures to properly determine the accuracy of the ML model and the utility of the model in real-world settings. Collecting large samples of social media data, particularly privately available data, in addition to users’ responses to validated clinical measures is a significant challenge for researchers. For example, a recent study using ML to predict eating disorder status (based on their responses to validated eating disorder screening surveys) from women’s internet browsing history only had 25% of the original sample agree to provide their internet data [48]. Encouragingly, participants in that study did not differ on any variables of interest (e.g., age, body mass index, eating disorder status) based on their willingness to provide their browsing history. More research is needed using ML to predict eating disorder status based on social media content, particularly using both image and text content as predictors and validated clinical measures as outcomes. However, current research suggests that social media and other online platforms may provide untapped potential to use ML to identify both those with a current eating disorder and those at risk of developing a disorder in the future on a mass scale.

With most personal data stored digitally, there is potential to combine multimodal data to improve the prediction of eating disorder risk. While little research has examined this potential, it is not a new line of inquiry. Over 20 years ago, researchers in Italy used ML to predict eating disorder status among a group of 172 patients with 87% accuracy based on 124 different variables, including generic information, ailment behaviour, blood samples, and psychodiagnostic testing [49]. The study had a small sample size, but it shows potential for the use of ML in this field. There have since been advances in technology that provide further opportunities to identify those at risk using more objective measures. For example, preliminary ML research has identified biomarkers of eating disorders from small samples of neuroimaging data (see Table 3 for a summary of current research using ML to identify eating disorder risk from physiological measures), with higher accuracy found among adult compared adolescent female samples [50–55]. Researchers are also examining potential endophenotypes that link specific genes to eating disorders [56], and have used ML to identify risk for anorexia nervosa with 69% accuracy using whole genome genotyping data [57]. Further, ML is currently being used to identify biomarkers for the effects of eating disorder treatment (i.e., cognitive behavioural therapy) longitudinally using neural circuit functioning, clinical data, gene expression, and psychological measures, but the results are yet to be published [58]. Thus, ML provides potential to examine complex interactions from many sources of data, such as neuroimages, genetics, social media, electronic health records, and sensor and usage data from smartphones or watches, to improve the accuracy of predictions of eating disorder risk.

**Table 3:** Research using ML to detect eating disorder risk via physiological predictors

Study	Sample	Predictors variables	Outcome variables	Best performing ML approach	Explanatory power of best performing model	Limitations
Cerasa et al. [50]	36 females, with an ED diagnosis (*n* = 17) or body mass index-matched healthy controls (*n* = 17)	Structural magnetic resonance images	ED status	Support vector machine	85%	Small sample size
Cyr et al. [52]	84 female adolescents, who met criteria for BN (*n* = 28), subclinical BN (SBN; *n* = 16), or healthy controls (HC; *n* = 40)	Functional magnetic resonance images of fronto-striatal regions during performance of a Simon task	ED status	Support vector machine	BN v. HC = 58% SBN v. HC = 64% Train BN v. HC, Test SBN v. HC = 66%	Small sample
Guo et al. [57]	13,206 adolescent and adult females, who have AN (*n* = 3940) or healthy controls (*n* = 9266)	Whole genome genotyping data	AN status	Logistic regression with LASSO penalty	69%	
Ioannidis et al. [70]	3937 observations from 36 AN inpatients	Physiological parameters, blood investigations over a 1-year period	Medical risk defined by independent clinical rates of deteriorating cases	Random forest	98%	Small sample
Lavagnino et al. [55]	30 females, with an AN diagnosis (*n* = 15) or demographically matched healthy controls (*n* = 15)	Structural neuroimaging scans	ED status	Least absolute shrinkage and selection operator (LASSO)	83%	Small sample
Lavagnino et al. [54]	67 adult females, who have restrictive-type AN (*n* = 19), who have recovered from restrictive-type AN (*n* = 24), or healthy controls (*n* = 24)	Structural brain scans to test cortical thickness	ED status	Linear relevance vector machine	AN v. HC = 74%	Small sample
Strigo et al. [53]	1 adolescent female with mixed ED, depressive, and gastrointestinal symptoms	Functional magnetic resonance images during a pain anticipation program and psychological survey responses created on previous samples	Which diagnostic phenotype most closely approximates the patient	Support vector machine	56% based on brain activation 84% based on psychological variables	Case study
Weygandt et al. [51]	70 females, who met criteria for binge eating disorder (BED; *n* = 17), or bulimia nervosa (BN; *n* = 14), or normal-weight controls (C-NW; *n* = 19) or overweight controls (C-OW; *n* = 17)	Functional imaging from a whole-body tomograph while viewing food or neutral images	ED status	Support vector machine	BED v. C-NW = 86% BN v. C-NW = 78% BED v. C-OW = 71% BN v. C-OW = 86% BED v. BN = 84%	Small sample size

ED, eating disorder; AN, anorexia nervosa; BN, bulimia nervosa; BED, binge eating disorder; HC, healthy controls; ML, machine learning

## Implications for early intervention and treatment

After identifying those at risk of eating disorders, there may be the opportunity to provide help seeking information to those individuals on social media or other online environments. Chatbots are conversational search assistants that often use ML to simulate modest conversation potentially employing basic therapeutic techniques and providing relevant referral information to healthcare providers [59]. While these chatbots would likely not replace the need for clinical interventions [59], they can provide basic help to those who need it, when they need it, anytime, anywhere, and for free. Stigma around eating disorders and weight is common [60], and may stop people from seeking help, particularly among groups for which eating disorders are less recognised (e.g., men, people of colour, higher-weight individuals). Chatbots may reduce stigma and discomfort with seeking help for an eating disorder and could be used to normalise a person’s symptoms and fear of disclosure before involvement from a clinician [61]. The use of chatbots in healthcare settings is just emerging, with most chatbots being text-based rather than speech-based, and most delivered via mobile apps [59]. Chatbots have recently been developed to provide specific text-based psychoeducation and coping skills training around body image and eating disorders [62, 63]. A recent study found that the use of a chatbot to administer an eating disorders prevention program to women at high risk of eating disorders reduced their weight/shape concerns through 6-month follow up and reduced their overall eating disorder psychopathology in the short term compared to waitlist controls [64]. Further research is needed on the efficacy and acceptability of chatbots in the field of eating disorders and the use of ML to increase the appropriateness of responses to specific and unanticipated user input [63]. However, there is potential for chatbots to be used on social media, and other platforms, to provide initial advice, prevention strategies, and support for individuals and carers, and to refer people to other effective evidence-based eating disorder prevention and treatment options.

There has recently been an increase in research testing the effectiveness of scalable online eating disorder treatments and interventions delivered via computers and mobile apps [5]. Like chatbots, online treatments and interventions can overcome some barriers to in-person help seeking because they can allow for anonymity, and have increased accessibility and flexibility [65]. A recent meta-analysis found online eating disorder prevention and treatment programs to have promising effects for reducing eating disorder symptoms immediately after the interventions, but more research is needed to determine their long term efficacy [5]. Smartphone interventions have also been created for eating disorders [66], with interventions using cognitive behavioural therapy being found to be effective at reducing eating disorder psychopathology compared to waitlist controls [67]. Online eating disorder programs often have issues with drop out (21–25%) and adherence [5], leading to a call for more engaging multimedia programs, codesign with end-users, and the use of ML to provide personalised approaches to interventions [5, 68]. While some human involvement may be optimal to provide more effective results and improve adherence [65], accessible and scalable effective evidence-based online eating disorder programs provide potential to reduce the prevalence and severity of eating disorders globally.

Researchers, clinicians, and those with lived experience are advocating for personalised approaches to eating disorder treatment [13, 69]. ML provides vast potential to tailor treatment plans to individuals in real time. ML could be used to help determine who will respond best to different in-person and online treatments and to track the progress of that treatment for individuals over time. For example, researchers have recently developed a ML early warning system that accurately identifies critically deteriorating cases in anorexia nervosa inpatient populations with 98% accuracy [70]. If a treatment is not working for an individual, ML could help decide which alternative treatment would be most beneficial. ML could also be used to match patients to specific therapists or support groups and to discover aspects of interactions between clinicians and patients or people within support groups that predict the best responses [23]. Further, ML may be a cost-effective tool to assess treatment fidelity with rapid, individualised, and objective feedback. A recent systematic review found that ML performed better than chance and, in some instances, at near human-level performance when predicting fidelity for psychological treatments from verbal recordings of treatment sessions [71]. More research is needed to explore the vast utility of ML in treatment settings for eating disorders.

Outside of the clinic, there is also potential for the use of ML in ecological momentary interventions (EMI; [72]) to deliver contextually relevant and personalised therapy recommendations based on survey and/or sensory data via smartphones, continually learning from the individual to identify risk early and provide immediate individualised care [61, 73]. Further, individuals, clinicians, and researchers could make use of self-quantification data for EMI, in which individuals track their own progress on different physical (e.g., heartrate, activity level, sleep) or mental health (e.g., mood, triggering experiences, eating disorder symptoms) domains via smartphones, to track each individual’s eating disorder risk in real time [74]. EMI with ML are currently being developed to improve eating behaviour based on ecological momentary assessments (i.e., completing brief surveys several times each day on mobile devices; 75). While EMI are being developed in the field of eating disorders, ML is yet to be utilised in these settings [5, 73, 76].

## Limitations

While ML provides exciting potential for the detection, prevention, and treatment of eating disorders, there are many factors that limit its current use in research and practice. Many of the limitations of ML are the same as those for traditional statistics. For example, large datasets with diverse participants are needed to create accurate ML models, which can be difficult to establish given the relatively low prevalence of eating disorders [2]. Further, those models need to be externally validated on large and diverse samples to ensure the predictions are generalisable to different populations. Current ML research in the field of eating disorders primarily focuses on samples of young white females, limiting the generalisability of existing models to detect risk in other relevant demographics. ML models are also limited by the quality of data used to create them and are susceptible to missing data. For example, if a model is created based on data from those seeking treatment for an eating disorder, it may not generalise to those with an eating disorder who do not seek help. Further, if supervised ML models are created to predict eating disorder status based on survey responses or clinical interviews, the accuracy of the models will only be as useful as those surveys/interviews are at capturing eating disorder status. Most ML models created in mental health have failed to generalise to diverse samples, which is a primary reason these models are not currently being used in practice [24]. Thus, there is currently a disconnect between what is possible and what is feasible in the field of ML and mental health. While these limitations can be overcome, it may require researchers to collaborate and combine large international datasets, especially if models are created to identify specific types of eating disorders (e.g., anorexia nervosa or bulimia nervosa). Best practice guidelines have been published for developing and reporting ML models in biomedical research [28]. These guidelines should be followed in future eating disorder research to ensure ML models are correctly applied and reported in this emerging field and to increase the utility of those models in real-world settings.

## Ethical considerations

If accurate and generalisable predictions can be made in the field of eating disorders, there are several ethical considerations that need to be addressed before using these methods in practice. There are concerns with the privacy and security of collecting, storing, and sharing data that may have implications for a person’s mental health. If ML models are created using digital data, there is also the concern about whether participants have, or need to, provide informed consent for their data to be used to make mental health predictions. More so if treatments are automated. Researchers are arguing for the need for people to understand, control, and own their own data and for that data to be stored securely [77].

There are also concerns around bias created in ML models that may disadvantage groups that are underrepresented in research. For example, if models are created using data from those who are seeking treatment for an eating disorder in a Western country, the models may assume that people with an eating disorder are young white females, and may not identify eating disorders among other genders, races, or ages. This bias could further perpetuate the problem by providing help seeking information only to those who match the demographic profile from the population in which the models were created. Researchers need to be conscious of, and reduce, any potential bias in ML models before they are used in practice. Bias may be reduced by creating ML models using large and diverse samples and by co-designing models with researchers, clinicians, and those with lived experience. Sharing de-identified data and computer code alongside the peer-reviewed publication of results can improve trust and transparency [78]. Like other technologies used in healthcare, ML algorithms should be transparent, rigorously tested, validated in real-world settings, and regularly reviewed if they are implemented in practice [78].

Like humans, all ML models have some degree of error, and that error could have harmful implications. For example, someone with an eating disorder may be missed by a ML model and they may not be provided with much needed help seeking information, or a person’s treatment may be stopped unnecessarily due to a prediction from a ML model. Identifying eating disorder risk based on digital data, such as that on social media, and providing help seeking information may also be distressing for those who were unaware of their vulnerability. Thus, researchers are advocating for the importance of collaboration between humans and ML algorithms in healthcare settings to make the most accurate and appropriate recommendations [79, 80]. Human involvement is needed because ML methods are not always transparent (i.e., it is sometimes unclear what predictions were based on), ML predictions can be inaccurate, and ML methods may not capture all of the intricacies of each specific situation [79]. Further, human involvement is important for therapeutic alliance which can improve treatment outcomes and can reduce higher dropout rates associated with completely remote care [81, 82]. However, it is important to note than human decision making in clinical settings can also be negatively impacted by conscious or unconscious biases [83, 84], which can lead to inaccurate diagnoses, unhelpful patient-clinician interactions, and inappropriate treatment recommendations. Thus, ML may be a useful tool to aid clinicians in the identification and treatment of eating disorders. Human-in-the-loop ML allows people to change the results of ML based on their skills and expertise, which can improve the power of ML to deal with complicated real-world tasks [79]. For example, ML models could be guided by researchers, clinicians, and those with lived experience to make recommendations based on available data, and then clinicians could decide whether to enact those recommendations based on their own clinical experience and knowledge.

## Practical considerations

There are also practical considerations when utilising ML in real-world settings. The models are reliant on the availability of the specific predictors used to create them. The more intricate, timely, and costly the predictors are to collect and input into the ML algorithms, the harder they will be to utilise in practice. For example, if ML models are reliant on genetic or neuroimaging data, they will be difficult and costly to use on a mass scale. Further, ML models that reply on predictors collected via technology (e.g., specific smartphones or smartwatches) will be limited to those who have access to that technology and/or those who choose to use that technology. Similarly, ML models that reply on social media data may only be useful for those who are active users of those specific platforms and those who engage in the specific social media behaviour (e.g., posting image or text content) used to create the ML models. Given the speed with which social media platforms and technology evolve to include new functions and features, ML models will need to be regularly reviewed and tested to ensure they remain accurate predictors of users’ eating disorder status. Further, those ML models may be reliant on companies (e.g., social media platforms) and users consenting for their information to be used to make predictions about mental health. Research on the opinions and concerns of companies and users regarding the use of their data to make mental health predictions is needed and education and interventions may be required before those ML models are used on a mass scale. Accurate ML models that reply on predictors that can be collected from the largest number of people with the least amount of burden (i.e., lowest cost and time) placed on individuals, researchers, clinicians, companies, and healthcare providers will likely be more widely used in practice.

Once the specific predictors have been collected, they will then need to be input into the ML algorithms. Ideally, this process would be automated. However, it may be difficult to automate if the ML models reply on predictors from different sources. If humans need to input data into the ML models, then simple interfaces should be created to allow them to enter the information without the need for extensive technical training. Further, in settings where clinicians and other healthcare providers need to decide whether to enact the recommendations from the ML models, the ML predictions need to be clearly presented, with an explanation of what the prediction was based on, if that information is available. Issues may also arise from a collaboration between clinicians and ML models. Clinicians may not value the recommendations made by ML models or they may rely solely on the recommendations at the expense of their own clinical judgement. Research and training would be required to ensure best practice in this space.

## Conclusions

Machine learning provides vast potential for the accurate, rapid, and cost-effective detection, prevention, and treatment of eating disorders. This potential is just beginning to be explored and more research is needed with large samples of diverse participants to ensure that ML models are accurate, unbiased, and generalisable to all people with eating disorders. There are important limitations and ethical and practical considerations with utilising ML methods in real-world settings. If accurate and generalisable ML models can be created in the field of eating disorders, especially using digital data, it has the potential to improve the way we identify, prevent, and treat eating disorders and could help reduce the prevalence and severity of these debilitating conditions.

## Data Availability

Not applicable.

## Abbreviations

EMI: Ecological momentary interventions
ML: Machine learning
